# Psychometric characteristics of DLQI-BRA and Skindex-16 to measure the impact of dermatological diseases on quality of life in Brazilian patients

**DOI:** 10.1371/journal.pone.0254882

**Published:** 2021-08-13

**Authors:** Silmara Meneguin, Ticiane Dionozio de Souza Matos, Camila Fernandes Pollo, Miriane Garuzi, Hélio Amante Miot, César de Oliveira

**Affiliations:** 1 Nursing Department, Botucatu Medical School, São Paulo State University, São Paulo, Brazil; 2 Program in Pathophysiology in Medical Practice, Botucatu Medical School, São Paulo State University, São Paulo, Brazil; 3 Department of Dermatology and Radiotherapy, São Paulo State University, Botucatu Medical School, São Paulo, Brazil; 4 Department of Epidemiology & Public Health, University College London, London, United Kingdom; International Medical University, MALAYSIA

## Abstract

**Objective:**

To compare the psychometric performance of the Dermatology Life Quality Index (DLQI-BRA) and Skindex-16 to assess quality of life (QoL) in Brazilian patients with dermatological diseases.

**Methods:**

This was a cross-sectional study carried out in a dermatology outpatient clinic of the São Paulo State University, with 188 patients with dermatological diseases. QoL was evaluated using the Dermatology Life Quality Index (DLQI-BRA) and Skindex-16. Cronbach’s alpha and Intraclass Correlation for Perfect Concordance (ICC) were used to analyse the reliability and temporal stability, respectively.

**Results:**

A positive correlation was found between the total Skindex-16 score and DLQI-BRA (0.75). Both instruments showed a significant (p< 0.01) reduction in their scores at the second assessment, demonstrating that they were sensitive in detecting changes in responsiveness in cases where there was a clinical change. Cronbach alpha coefficients for the instruments showed satisfactory performance (>0,7), but Skindex-16 displayed the highest Cronbach alpha (0.94; CI = 0.93–0.95).

**Conclusion:**

Both instruments tested showed a good psychometric performance assessing QoL in patients with skin dermatoses. The instruments displayed reliability and temporal stability as well as responsiveness.

## Introduction

Many dermatological diseases are not directly life threatening, but can cause great physical and psychological discomfort by affecting one’s body image perception and causing uncomfortable symptoms such as pain, itching and burning that in turn can have a negative impact on patients’ perception of quality of life (QoL) [1]. It is estimated that at least one third of patients with skin diseases suffer emotional distress associated with their dermatosis [2]. Previous studies have shown that low morbidity dermatoses impair self-image and can lead to depression and anxiety as well as serious systemic diseases and they can seriously affect an individual’s psychological, work and social functions [3, 4].

In the literature, the instruments for evaluating QoL related to skin disorders that stand out are the Dermatology Life Quality Index (DLQI-BRA) and the Skindex-16 [5]. DLQI-BRA has the advantage of being widely used by dermatologists and researchers [6]. It is very easy to apply as it has only ten items. However, its questions focus mainly on the physical limitations with only few items assessing the psychological impact of skin diseases [7]. Skindex-16 is a more recent instrument that has been less used in clinical studies. Derived from Skindex-29, the questionnaire consists of 16 items and is easy to apply [7, 8]. It focuses more on psychological issues, which can be particularly important as skin disorders can have a major impact on psychological aspects [9]. A translated Brazilian Portuguese version of Skindex-16 is already available [10].

Neither DLQI-BRA nor Skindex-16 are considered the gold standard for evaluating QoL in patients with skin conditions. Furthermore, there is no consensus on which instrument should be used to evaluate the impact of a specific skin disease or change on QoL. Therefore, health professionals must adopt the most appropriate instrument for their patients.

In the last decade, skin diseases have begun to be systematically evaluated. Since then, there has been a greater interest in developing methods to measure QoL in dermatology, given that QoL is dependent on psychometric instruments [10, 11]. In this context, psychometry is one of the ways of taking measurements using tests which estimate constructs (also known as latent variables) which are characteristics of individuals which cannot be directly observed. According to the Consensus-based Standards for the Selection of Health Measurement Instruments (COSMIN), an instrument structure has three pillars: reliability, validity and responsiveness [12–14].

There is still a clear need in the literature for comparative studies analysing the psychometric performance of dermatological instruments used to assess QoL. Our study takes into account that skin diseases are complex and affect the physical, psychological and social areas of life which in turn can impact on QoL and coping strategies. In this sense, the Skindex-16 may have a better psychometric performance as it addresses other QoL domains, not just the physical domain as the DLQI-BRA. Therefore, the present investigation aimed to compare the psychometric performance of DLQI-BRA and Skindex-16 in assessing the quality of life of Brazilian patients with dermatological diseases.

## Methods

This was a cross-sectional study conducted with 188 patients with skin diseases at the dermatology outpatient clinic of the São Paulo State University (UNESP) medical school, Botucatu, Brazil.

The following eligibility criteria were adopted: patients with skin diseases confirmed by dermatologists, members of the Brazilian Society of Dermatology, and documented in medical records; aged 18 or older, from both sexes and who consented to participate. Patients who were not available or unable to complete the interview were excluded.

Data were collected between March 2018 and March 2019 using a questionnaire divided into three parts: first, socio-demographic and clinical data; second, QoL assessment using DLQI-BRA (Dermatology Life Quality Index) and Skindex-16.

The DLQI-BRA is a single domain instrument composed of 10 items, divided into six aspects: symptoms and feelings, daily activities, leisure, work/school, personal relationships and treatment. Its scores range from 0 to 30 with 0–1 = having no impact; 2–5 = light impact; 6–10 = moderate impact; 11–20 = substantial impact and 21–30 = extreme impact on patient’s QoL. The higher the score, the worse the quality of life [15].

The Skindex-16 is a multidomain instrument in which answers are given on a 7-point Likert scale, ranging from 0 (never bothered) to 6 (always bothered), according to the frequency with which the patient was worried by their skin condition over the previous seven days. It consists of three domains: symptoms (items 1 to 4), emotions (items 5 to 11), and functionality (items 12 to 16). All responses are transformed on a linear scale ranging from 0 to 100 points. Scores for each of the three domains (symptoms, emotions and functionality) are calculated. The higher the value found, the worse the quality of life [10].

Quantitative variables were analysed in terms of means and standard deviations, while classification variables were presented in Tables as absolute numbers and their relative (%) frequencies. The QoL instruments’ scores were tested for correlation using the Spearman’s coefficient test.

### Internal consistency

The internal consistency of each of the Skindex-6 domains was assessed using the Cronbach’s alpha and values between 0.70 and 0.95 were considered acceptable [16].

### Convergent validity

The relationships between Skindex-16 domains and DLQI-BRA were assessed using the Spearman’s correlation coefficient test. A value of rho>0.6 was expected for the correlation between the Skindex-16 domains (symptoms, emotions and functioning) and the DLQI-BRA [17].

### Temporal stability and responsiveness

Forty patients were re-assessed between 7 and 14 days after their first interview by the same investigator. The temporal stability was assessed in cases that did not show any clinical change in their disease status. The instrument’s stability was investigated using the intra-class correlation coefficient (ICC), with values greater than 0.7 being considered acceptable (CI = 95%). Responsiveness was assessed in cases where patients showed clinical improvements using the Wilcoxon test [18].

Our sampling was defined to satisfy a multivariate model, according to Freeman’s formula [19].

The statistical analyses were performed using SPSS version 25® software (mIRT package). The research project was approved by the Ethics Committee of the São Paulo State University (UNESP) medical school (protocol n^o^ 2.392.601). All of the patients were informed of the benefits and risks related to the study and provided their written informed consent for the study and for the publication of results.

## Results

### Sample characteristics

The final analytical sample comprised of 188 patients with skin disease. Overall, there were 95 male participants (51%), 69% living with a partner, 54% with a lower educational level and the mean age was 55 years. More than two thirds of the sample had an average monthly family income below R$3,000.00 ($552.00). In relation to the QoL assessment, the instruments showed that the impact of skin disease on participants’ quality of life was moderate. The median QOL scores were as follows: DLQI-BRA = 8 (4–13), Skindex-16s = 46 (21–63), Skindex-16e = 55 (21–74) and Skindex-16f = 32 (05–60), respectively (Table 1).

**Table 1:** Social demographic characteristics and quality of life of participants (n = 188).

Variable	N (100%)
**Age (years)** *	55 (16)
**Gender**	
Female	93 (49)
Male	95 (51)
**Marital Status**	
Living with a partner	129 (69)
Single	59 (31)
**Education**	
Elementary	102 (54)
High School	65 (35)
Further	2 (11)
**Monthly Family Income**	
< R$1,000.00	21 (11)
R$1,100.00 to R$ 2,999.00	106 (56)
R$ 3,000.00 to R$5,000.00	45 (24)
**DLQI-BRA** **	8 (4–13)
**Skindex-16 S** **	46 (21–63)
**Skindex-16 E** **	55 (21–74)
**Skindex-16 F** **	32 (05–60)

* Mean (Standard Deviation)

** Median (p25-p75); Skindex-16 symptoms; Skindex-16 emotions; Skindex-16 functionality.

Table 2 shows the median quality of life scores for all dermatological conditions included in the study. The most prevalent clinical condition was psoriasis (53%), followed by cellulitis/erysipelas (22.3%), chronic ulcers (5.3%) and eczematous dermatosis (7.9%). Other dermatoses accounted for 11%.

**Table 2:** Median quality of life scores for each dermatological condition included in the study (n = 188).

		DLQI-BRA	SK- 16S	SK -16E	SK- 16F
	SAMPLE	MEDIAN	MEDIAN	MEDIAN	MEDIAN
Psoriasis	100	5,70	40,62	40,76	26,07
Erysipelas	32	13,00	67,98	65,63	53,54
Dermatitis Contact	10	13,40	51,25	56,17	43,33
Ulcer	10	10,50	45,83	65,71	42,00
Cellulite	10	11,80	46,66	59,52	47,32
Dermatitis Atopic	5	6,50	56,25	51,79	29,16
Melanomas	4	7,25	16,68	20,85	25,03
Carcinoma Squamous Cell	2	9,00	20,85	20,25	51,65
Leprosy	2	10,00	14,60	69,05	48,35
Pressure Ulcer	2	17,00	62,50	76,20	76,65
Vitiligo	2	7,50	6,25	66,70	26,70
Histoplasmosis	1	5,00	41,70	57,10	0,00
Ichthyosis	1	6,00	50,00	31,00	53,30
Leishmaniasis	1	15,00	33,30	88,10	63,30
Mycosis Fungoides	1	25,00	66,70	81,00	76,70
Pemphigus	1	26,00	58,30	69,00	100,00
Pityriasis Versicolor	1	0,00	0,00	26,20	0,00
Keratoderma Palmoplantar	1	4,00	8,30	2,40	3,30
Urticaria	1	16,00	58,30	45,20	50,00
Alopecia	1	13,00	58,30	100,00	70,00

### Internal consistency

Table 3 shows the Cronbach alpha coefficients for the instruments and their respective domains. Both instruments showed satisfactory performance i.e. α > 0.7. Skindex-16 displayed the highest Cronbach alpha (0.94; CI = 0.93–0.95).

**Table 3:** Internal consistency (Cronbach alpha) for DLQI-BRA, and SKINDEX-16 and its respective domains (n = 188).

Instrument	Cronbach α (IC 95%)
DLQI-BRA	0.85 (0.82–0.88)
Skindex-16	0.94 (0.93–0.95)
Skindex-16 s *	0.79 (0.73–0.83)
Skindex-16 e **	0.92 (0.90–0.94)
Skindex-16 f ***	0.92 (0.89–0.93)

* Skindex-16 symptoms

** Skindex-16 emotions

*** Skindex-16 functionality.

### Convergent validity

Table 4 displays the correlations between DLQI-BRA, total Skindex-16 score and its domains (Sk s = symptoms, Sk e = emotions, Sk f = functionality) scores. High correlations can be seen between total Skindex-16 score and DLQI-BRA (0.75).

**Table 4:** Spearman coefficients of correlation (ρ) between SKINDEX-16 and its domains, and DLQI-BRA (n = 188).

	Skindex-16 Total	Sk-16 s	Sk-16 e	Sk-16 f
DLQI-BRA	0.75	0.57	0.66	0.70
Skindex-16 Total		0.77	0.93	0.87
Sk-16 s			0.62	0.54
Sk-16 e				0.71
Sk-16 f				

All correlations result in p<0.01.

Sk-16 s—Skindex-16 symptoms; Sk-16 e—Skindex-16 emotions; Sk-16 f—Skindex-16 functionality.

### Responsiveness

Table 5 shows the median values for the total DLQI-BRA score and Skindex-16 domains in the two occasions when responsiveness was assessed. Both instruments showed a significant (p< 0.01) reduction in their scores at the second assessment, demonstrating that both instruments were sensitive in detecting changes in responsiveness in cases where there was a clinical change.

**Table 5:** Responsiveness distribution for DLQI-BRA and Skindex-16 domains.

	M1*	M2**	p-value
DLQI -BRA***	10 (6.5–15.5)	7.50 (4.5–13)	<0.01
Sk-16 s***	9.5 (5–13)	7 (3.5–10)	<0.01
Sk-16 e***	25.5 (9.5–30.5)	19 (6–25.5)	<0.01
Sk-16 f***	15 (6.5–20)	9 (5–15)	<0.01

Sk-16 s—Skindex-16 symptoms; Sk-16 e—Skindex-16 emotions; Sk-16 f—Skindex-16 functionality.

* Moment 1 –first interview

** Moment 2 –second interview after 7 to 14 days

*** Median (p25-p75).

### Test-retest reliability

Table 6 shows the median values for the total DLQI-BRA score and the Skindex-16 domains in the two moments when temporal stability was assessed. There was little change in the second assessment score (ICC > 0.7) as there was no clinical change.

**Table 6:** Temporal stability distribution for DLQI-BRA and the Skindex-16 domains.

Test-retest	M1*	M2**	ICC****
DLQI-BRA***	9 (4.5–11)	10 (5.5–11.5)	0.95 (0.88–0.98)
Sk-16 s***	10.5 (7.5–15)	11(7.5–16.5)	0.98(0. 96–0.99)
Sk-16 e***	21 (10–27)	21 (9.5–28)	0.96 (0.91–0.98)
Sk-16 f***	11.5 (5–16)	12(4.5–16.5)	0.95 (0.90–0.98)

Sk-16 s—Skindex-16 symptoms; Sk-16 e—Skindex-16 emotions; Sk-16 f—Skindex-16 functionality.

* Moment 1 –first interview

** Moment 2 –second interview after 7 to 14 days

*** Median (p25-p75)

**** Intraclass correlation coefficient (ICC).

## Discussion

The main findings of this study that assessed the psychometric performance of DLQI-BRA and Skindex-16 to measure the impact of dermatological diseases on quality of life in Brazilian patients, showed that both instruments have a good psychometric performance assessing QoL in patients with skin dermatoses. In addition, the multidimensional nature of Skindex-16 allows the identification of impairment in other domains compared to DLQI-BRA that focuses on physical impairments.

In dermatology, QoL should be evaluated during routine consultations as many conditions and problems can be treated or even resolved when individuals feel they were listened to, understood and respected [20]. In this sense, it is important to take a deeper look at these patients since their dermatological problems are often neglected for being judged as non-fatal. Therefore, QoL studies have become increasingly relevant, as they can reveal how much dermatoses affect a patient’s daily life. Skin diseases are extremely common and personally, socially, and professionally affect between 30 and 70% of the world population [19, 20].

In this QoL investigation, both DLQI-BRA and Skindex-16 revealed a moderate level of impact of skin disease on participants’ QoL, despite both instruments having different ways of evaluating the construct. When choosing an instrument for assessing QoL, a researcher should ensure the reliability of their findings. Before its application, it is essential to know the items, their domains, the forms of evaluation, and, especially, the measurement properties. These aspects are crucial since the quality of the information provided by the instrument depends, in part, on a good psychometric performance [21, 22].

When an instrument has good psychometric performance, it means that it possesses certain characteristics that attest to its reliability, with validity and precision [23, 24]. In the present study, both instruments presented satisfactory reliability with values over 0.9 for the three Skindex-16 domains and of 0.8 for DLQI-BRA.

A recent Brazilian study which evaluated Skindex-16 presented similar Cronbach alpha values to those found in our investigation [10] (-). Another study with 548 patients showed that DLQI-BRA had a higher level of reliability for clinical changes compared to other dermatology measuring instruments, with a Cronbach alpha of 0.85 [25].

However, it should be noted that reliability is not a fixed property, as it varies according to circumstances, population and type of study. Considering that measurement instruments integrate clinical practice and research in different areas of knowledge, assessing their reliability is essential in selecting instruments that provide valid and reliable measurements [24].

According to the correlation coefficient analysis, our findings showed that DLQI-BRA, globally the most common instrument for evaluating QoL in dermatology, strongly correlated (>0.7) with Skindex-16 and with the Skindex-16 functional domain. The Skindex-16 symptom and emotional domains presented moderate correlation (0.57 and 0.66, respectively). This could be attributed to the fact that DLQI-BRA focuses mainly on the physical QoL aspects. These findings are in agreement with another Brazilian study in which the coefficient of correlation values were considered clinically significant in relation to Skindex-16 domains and DLQI-BRA i.e. greater than 0.6 [10].

The results found in our study suggest a positive correlation between the domains that deal with emotional and functional issues in dermatological instruments. We showed that Skindex-16 presented higher sensitivity than DLQI-BRA, in addition to the physical aspect, since it identified impairment in the other QoL domains. A study performed at the Barretos Hospital, Sao Paulo, Brazil with 161 patients showed that Skindex-16 is a valid and reliable instrument for evaluating the implications of dermatological conditions on QoL in a Brazilian population [10].

With regards to instrument responsiveness, applying the instruments for a second time in patients who presented clinical changes allowed us to show that the instruments scores also varied according to the change in dermatosis status with a significant reduction in score at the second moment (p <0.01).

We found a small change in scores related to temporal stability during the second interview i.e. ICC > 0.7, as there was no clinical change. This allowed us to say that the instrument obtained satisfactory results and is reproducible in clinical practice with the current analysed data. It is important to highlight that this study included patients with some of the most prevalent skin diseases in Brazil according to a recent study by the Brazilian Dermatology Society (BDS) [26].

A potential limitation of the study relates to its generalizability since psoriasis and erysipelas represented most of the dermatological conditions (70.2%).

Finally, it is important to highlight that the lack of similar studies on quality-of-life instruments in dermatology made it difficult for us to compare and discuss our results. However, it also showed that further studies are needed in this area. Ultimately, this study could help to identify the best generic instrument to assess QoL in patients with dermatological diseases and guide care and treatment strategies towards implementing actions based on interdisciplinary care focused on the real needs of these patients, which often go unnoticed.

## Conclusion

Both instruments tested showed a good psychometric performance to assess QoL in patients with skin dermatoses. The multidimensional nature of Skindex-16 was able to identify impairment in other quality of life domains in addition to the physical aspect of the DLQI-BRA.

The instruments displayed reliability and temporal stability as well as responsiveness. They were able to detect changes in QoL in cases where there was a clinical modification of the dermatological disease.

## Supporting information

S1 Checklist (DOCX)

S1 Data (XLSX)
